# Carfilzomib enhances cisplatin-induced apoptosis in SK-N-BE(2)-M17 human neuroblastoma cells

**DOI:** 10.1038/s41598-019-41527-0

**Published:** 2019-03-25

**Authors:** Song-I Lee, Yeon Ju Jeong, Ah-Ran Yu, Hyeok Jin Kwak, Ji-Young Cha, Insug Kang, Eui-Ju Yeo

**Affiliations:** 1 Department of Health Sciences and Technology, GAIHST, Gachon University, Incheon, 21999 Republic of Korea; 2 Department of Biomedical Sciences, Graduate School, Kyung Hee University, Seoul, 02447 Republic of Korea; 3 Department of Biochemistry, College of Medicine, Gachon University, Incheon, 21999 Republic of Korea; 4 Department of Biochemistry and Molecular Biology, School of Medicine, Biomedical Science Institute, Kyung Hee University, Seoul, 02447 Republic of Korea

## Abstract

Neuroblastoma is a solid malignant tumor of the sympathetic nervous system, which accounts for 8–10% of childhood cancers. Considering the overall high risk and poor prognosis associated with neuroblastoma, effective therapeutics should be developed to improve patient survival and quality of life. A recent study showed that a proteasome inhibitor, carfilzomib (CFZ), reduced cell viability of SK-N-BE(2)-M17 neuroblastoma cells. Therefore, we investigated the molecular mechanisms by which CFZ lower the cell viability of neuroblastoma cells. CFZ reduced cell viability via cell cycle arrest at G2/M and apoptosis, which involved caspase activation (caspases-8, 9, 4, and 3), endoplasmic reticulum stress, reactive oxygen species production, mitochondrial membrane potential loss, and autophagy in a dose- and time-dependent manner. The effect of CFZ was additive to that of cisplatin (Cis), a well-known chemotherapeutic drug, in terms of cell viability reduction, cell cycle arrest, and apoptosis. Importantly, the additive effect of CFZ was maintained in Cis-resistant neuroblastoma cells. These results suggest that CFZ can be used in combination therapy for patients with neuroblastoma to overcome the resistance and adverse side effects of Cis.

## Introduction

Neuroblastoma originates from undifferentiated multipotent migratory neural crest cells in the sympathetic nervous system, adrenal medulla, or paraspinal ganglia^1^, and is known to be the most common extracranial solid cancer in infants and children^2^. More than 90% of the total incidence of neuroblastoma occurs before the age of 10 years^2,3^. Furthermore, neuroblastoma accounts for approximately 15% of childhood cancer-related mortality^4,5^. Despite the development of many new therapies for neuroblastoma, the overall survival rate for patients, especially children with high-risk (relapsed or metastatic) neuroblastoma, remains poor^2,6^. Therefore, more effective regimens with acceptable toxicity are required for patients with high-risk neuroblastoma^7^.

Carfilzomib (CFZ), a cell-permeable tetrapeptide epoxyketone analog of epoxomicin^8^, is a second-generation proteasome inhibitor that selectively and irreversibly binds to its target: the chymotrypsin-like subunit of proteasome^9^. CFZ has been developed as a drug with lesser toxic side effect than bortezomib (BZ) that is a first-generation proteasome inhibitor and has been approved by the Food and Drug Administration (FDA) of the United States for the treatment of patients with relapsed or refractory multiple myeloma^10^. Since CFZ has also been approved by the FDA for the treatment of multiple myeloma^11^, the antitumor effect of CFZ has been tested in several cancer cells^12–14^. Although accumulation of unfolded proteins, production of reactive oxygen species (ROS), induction of apoptosis and autophagy, cell cycle arrest, induction of pro-apoptotic proteins, and inhibition of the pro-survival signal pathways have been suggested as molecular mechanisms of CFZ action, the actual mechanism utilized depends on the cell types.

Accumulation of unfolded proteins can initially cause unfolded protein response (UPR), followed by abnormal ER function, finally resulting in ER stress and apoptosis^15,16^. In humans, caspase-4 is the initiator caspase for ER stress-mediated apoptosis. The UPR consists of three signaling branches: PERK–eIF2α, IRE1α–XBP1, and ATF6α^17,18^. The activated serine/threonine kinase PKR-like ER kinase (PERK) phosphorylates and inactivates eukaryotic initiation factor 2α (eIF2α), resulting in translation inhibition. The phosphorylated eIF2α selectively enhances the translation of activating transcription factor 4 (ATF4) mRNA, which up-regulates CCAAT-enhancer-binding protein homologous protein (CHOP)^19^. The activated IRE1α cleaves X-box binding protein 1 (XBP-1), and the cleaved XBP-1 (s-XBP1) moves to the nucleus and promotes the expression of ER chaperones, including glucose-regulated protein 78 (GRP78), GRP94, and CHOP^20,21^. ATF6α is cleaved at sites 1 and 2 by proteases in the Golgi apparatus, which acts as a transcription factor to regulate the expression of ER stress-associated genes, including *CHOP*.

ER stress cross-talks with mitochondrial dysfunction, ROS accumulation, and autophagy, which might regulate intrinsic and extrinsic apoptosis pathways^22,23^. Mitochondrial membrane damage and excessive ROS production can cause the intrinsic pathway via caspase-9 activation^24,25^, whereas death receptor activation and death-inducing signal complex formation can cause the extrinsic pathway via caspase-8 activation^26^. Thereafter, activated caspase-8 and -9 activate effector caspases, such as caspase-3 and 7, resulting in nuclease activation and apoptotic cell death^27–29^. Since CFZ induced apoptosis in SK-N-BE(2)-M17 neuroblastoma cells, the molecular mechanisms of CFZ action was further investigated in this cell type.

Cisplatin (Cis) is a platinum coordination compound, which is one of the most effective drugs used in chemotherapy^30^. Cis causes DNA damage via crosslink with the purine bases and inhibits DNA synthesis, mitosis, and DNA repair. Cis induces apoptosis through various pathways, such as death receptor signaling, activation of mitochondrial pathways, and generation of ROS, thereby damaging tumor cells. In addition, oxidative stress resulting from ROS production leads to p53 signaling and cell cycle arrest at the S phase. These apoptosis- and cell cycle arrest-inducing mechanisms underline the anticancer activity of the drug^31,32^. However, Cis chemotherapy is accompanied by drug resistance and various toxic side effects, including hepatotoxic, neurotoxic and nephrotoxic^30,33^. Therefore, it has been used in combination with other anticancer drugs for treating neuroblastoma, which are more effective and generate lesser side effects than the use of very high doses of Cis alone^30,34^. Therefore, in this study, we investigated whether CFZ can be utilized in combination with Cis and whether CFZ can exert a chemosensitization effect in Cis-resistant neuroblastoma cells.

## Results

### CFZ reduces cell viability in SK-N-BE(2)-M17 human neuroblastoma cells

**Figure 1:**
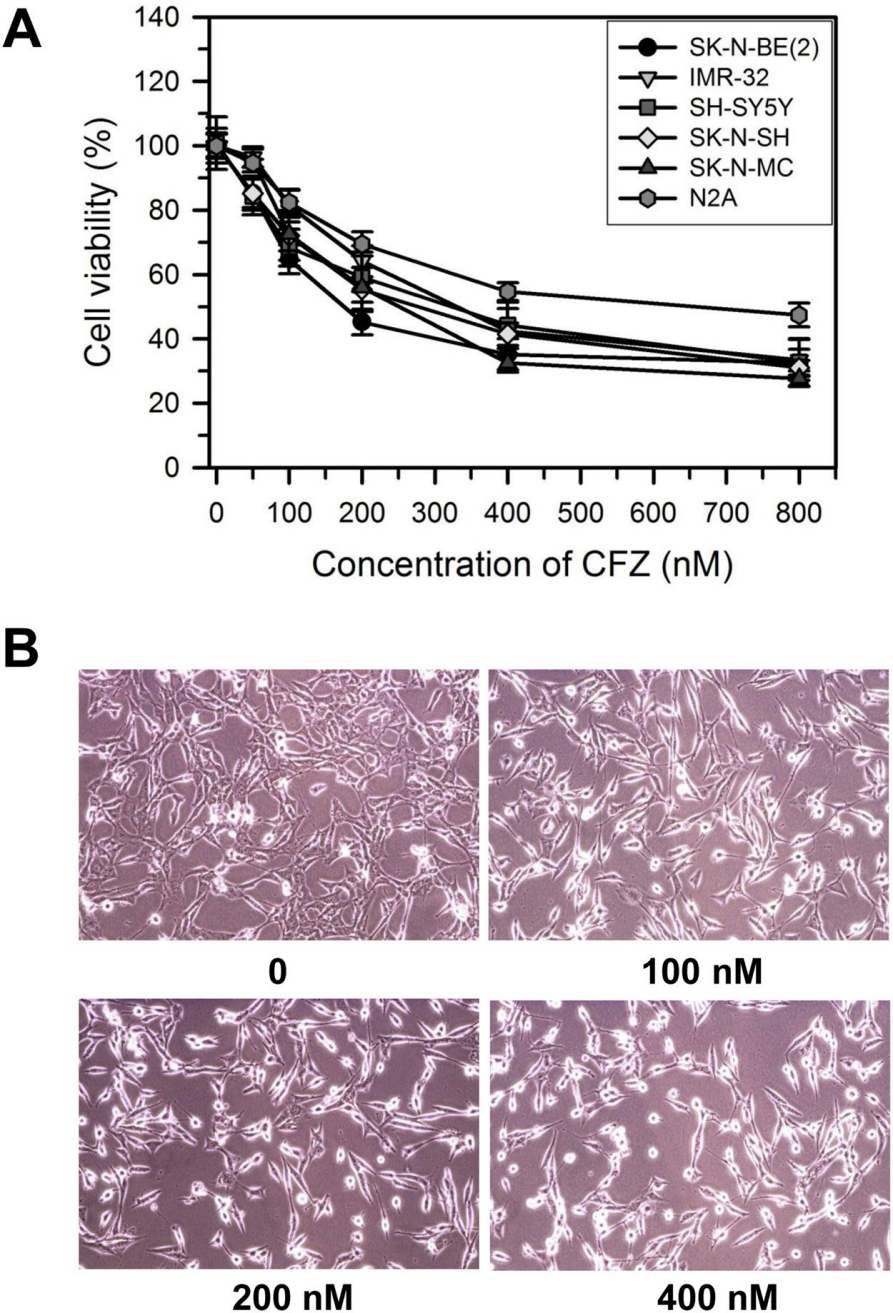
Effect of CFZ on cell morphology and viability of SK-N-BE(2)-M17 cells. (**A**) SK-N-BE(2)-M17, IMR-32, SH-SY5Y, SK-N-SH, SK-N-MC, and Neuro-2A (N2A) cells were treated with vehicle or various concentrations of CFZ for 24 h. Cell viability was assessed by the MTT assay. The percentages of cell viability are plotted as the mean ± standard deviation of at least three experiments. All data points are statistically (P < 0.05) significant compared to the vehicle-treated control (not shown). (**B**) Representative photomicrographs showing morphological changes in SK-N-BE(2)-M17 cells treated with vehicle (DMSO) or various concentrations (100–400 nM) of CFZ for 24 h.

The effect of CFZ on cell viability was determined in various neuroblastoma cells, including SK-N-BE(2)-M17 (male), IMR-32 (male, neuroblast, fibroblast, monolayer), SH-SY5Y (female, neuroblastoma), SK-N-SH (female, brain epithelial, monolayer), and SK-N-MC (female, brain epithelial, monolayer) human neuroblastoma cells, and Neuro-2A mouse neuroblastoma cells. Cells were treated with vehicle (DMSO) or various concentrations (50–800 nM) of CFZ for 24 h in DMEM containing 10% FBS and antibiotics/antimycotics. The cell viability was investigated by the MTT assay. Results showed that CFZ treatment decreased the cell viability of neuroblastoma cells in a dose-dependent manner (Fig. 1A). The IC_50_ values of CFZ for inhibition of SK-N-BE(2)-M17, IMR-32, SH-SY5Y, SK-N-SH, SK-N-MC, and Neuro-2A cells at 24 h are 204, 363, 287, 273, 260, and 607 nM, respectively. In the present study, we found that CFZ is most effective for reduction of cell viability in SK-N-BE(2)-M17 cells compared to other neuroblastoma cells. Human neuroblastoma cells can be discriminated by *MYCN* amplification: SK-N-BE(2)-M17 and IMR32 cells are *MYCN*-amplified but SH-SY5Y, SK-N-SH, and SK-N-MC cells are non-*MYCN*-amplified cells. CFZ was effective to both *MYCN*-amplified and non-*MYCN*-amplified neuroblastoma cells with slight differences in IC_50_ values in our experimental condition. Nevertheless, since about 25% of human neuroblastomas showed *MYCN*-amplification, which is associated with poor prognosis, SK-N-BE(2)-M17 cell line has been used as a model for the most aggressive and high-risk neuroblastoma. For these reasons, we concentrated on SK-N-BE(2)-M17 cells for the present study. Morphological changes of SK-N-BE(2)-M17 cells were examined after incubation with various concentrations of CFZ for 24 h. Changes in cell shape and detachment of cells were clearly visible after treatment with 100–400 nM of CFZ (Fig. 1B).

### CFZ induces cell cycle arrest and apoptotic cell death in SK-N-BE(2)-M17 cells

**Figure 2:**
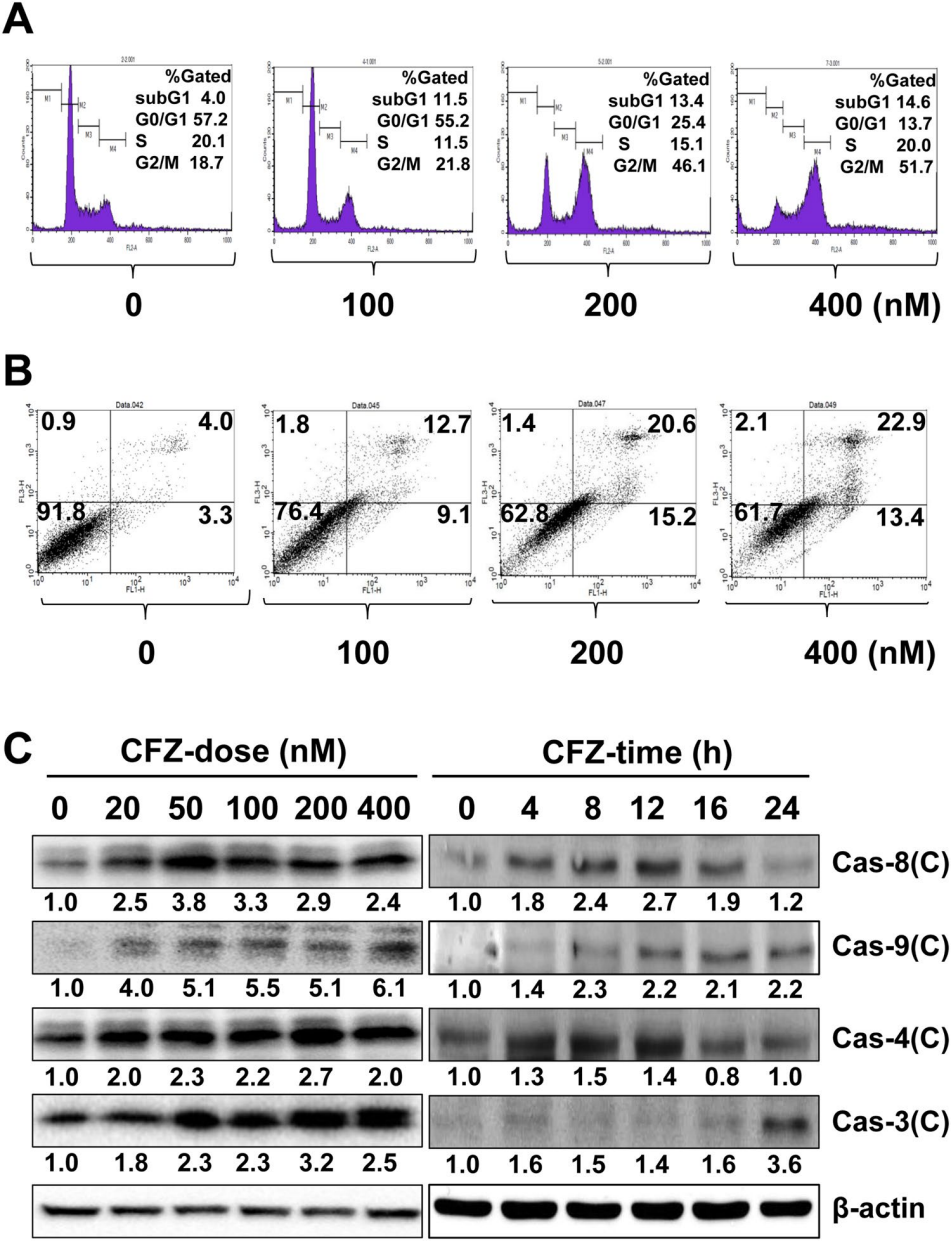
Effect of CFZ on cell cycle arrest and apoptosis in SK-N-BE(2)-M17 cells. (**A**) SK-N-BE(2)-M17 cells were treated with vehicle or 100–400 nM CFZ for 24 h and then stained with PI and analyzed by flow cytometry. The percentage of gated cells in the subG1, G0/G1, S, and G2/M areas is shown in the upper right area of each plot. (**B**) Cells were treated with vehicle or 100–400 nM CFZ for 24 h. Vehicle- or CFZ-treated cells were stained with PI and annexin V-FITC and evaluated by FACS analysis. The lower right and upper right areas represent the percentages of early apoptotic cells and late apoptotic cells, respectively. The fraction of necrotic cells is shown in the upper left area. **(C)** Cells were treated with various concentrations (20–400 nM) of CFZ for 24 h (CFZ-dose) or treated with 200 nM CFZ for the indicated durations (CFZ-time). Cells were then lysed and total cell extracts were resolved by SDS-PAGE. The levels of activated caspases were detected by western blot analysis using antibodies against cleaved forms of caspases (Cas-8, 9, 4, and 3), and β-actin (internal control). Blots are representative of those obtained in more than three independent experiments.

To determine whether the CFZ-induced cell viability reduction is due to cell cycle arrest or cell death, CFZ-treated cells were stained with PI and analyzed for cell cycle and DNA fragmentation by flow cytometry. Cells were treated with CFZ for 24 h. Results showed that the number of cells in the G2/M fraction increased from 18.7% to 21.8%. 46.1% and 51.7% after treatment with 100, 200 and 400 nM CFZ, respectively (Fig. 2A). At these concentrations of CFZ, the number of cells with fragmented DNA also increased from 4.0% to 11.5%, 13.4% and 14.6% at 100, 200, and 400 nM CFZ, respectively, as estimated from the increase in the subG1 population.

Since DNA fragmentation is one of the characteristics of apoptotic cell death, the effect of CFZ on apoptosis in SK-N-BE(2)-M17 cells was further examined using the PI/annexin V-FITC double staining and flow cytometry. Cells were treated with various concentrations of CFZ for 24 h and double-stained with PI and annexin V-FITC, and fluorescence intensity was detected by flow cytometry. The portions of early apoptotic (annexin V-positive/PI-negative, the lower right quadrant), late apoptotic (annexin V-positive/PI-positive, the upper right quadrant), and necrotic cells (annexin V-negative/PI-positive, the upper left quadrant) were compared with that of live cells (annexin V-negative/PI-negative, the lower left quadrant). As shown in Fig. 2B, CFZ treatment for 24 h increased the portion of early apoptotic cells from 3.3% to 9.1% at 100 nM, 15.2% at 200 nM and 13.4% at 400 nM. The ratio of late apoptotic cells also increased from 4.0% to 12.7%, 20.6%, and 22.9% after treatment with 100, 200, and 400 nM CFZ, respectively. These results suggest that CFZ reduced cell viability through induction of cell cycle arrest at G2/M and apoptotic cell death in SK-N-BE(2)-M17 cells.

An increase in the levels of cleaved caspases indicates the activation of caspases and apoptosis^35^. Therefore, the dose- and time-dependent changes in cleaved caspase levels (8, 9, 4, and 3) were investigated by western blot analysis. We observed that most caspases were activated by various concentrations (20–400 nM) of CFZ in a dose-dependent manner (Fig. 2C). The time-dependent activation of caspases were examined at a given, not toxic, dose near the IC_50_ value (200 nM). Caspases-8 and 4 were activated by 200 nM CFZ at an early time point (4 h), which was sustained until 12 h and then declined gradually (Fig. 2C). In contrast, caspase-9 and the final effector caspase-3 were activated at late time points (16–24 h) after treatment with 200 nM CFZ.

Since caspases-8 and 9 are activated, we suggest that CFZ can induce apoptosis via both intrinsic and extrinsic pathways. In addition, CFZ activated caspase-4, which is located in the ER membrane and associated with ER stress-induced cell death^36^, suggesting that ER stress may be involved in CFZ-induced apoptosis in SK-N-BE(2)-M17 cells.

### CFZ induces the expression and activation of ER stress-associated proteins in SK-N-BE(2)-M17 cells

**Figure 3:**
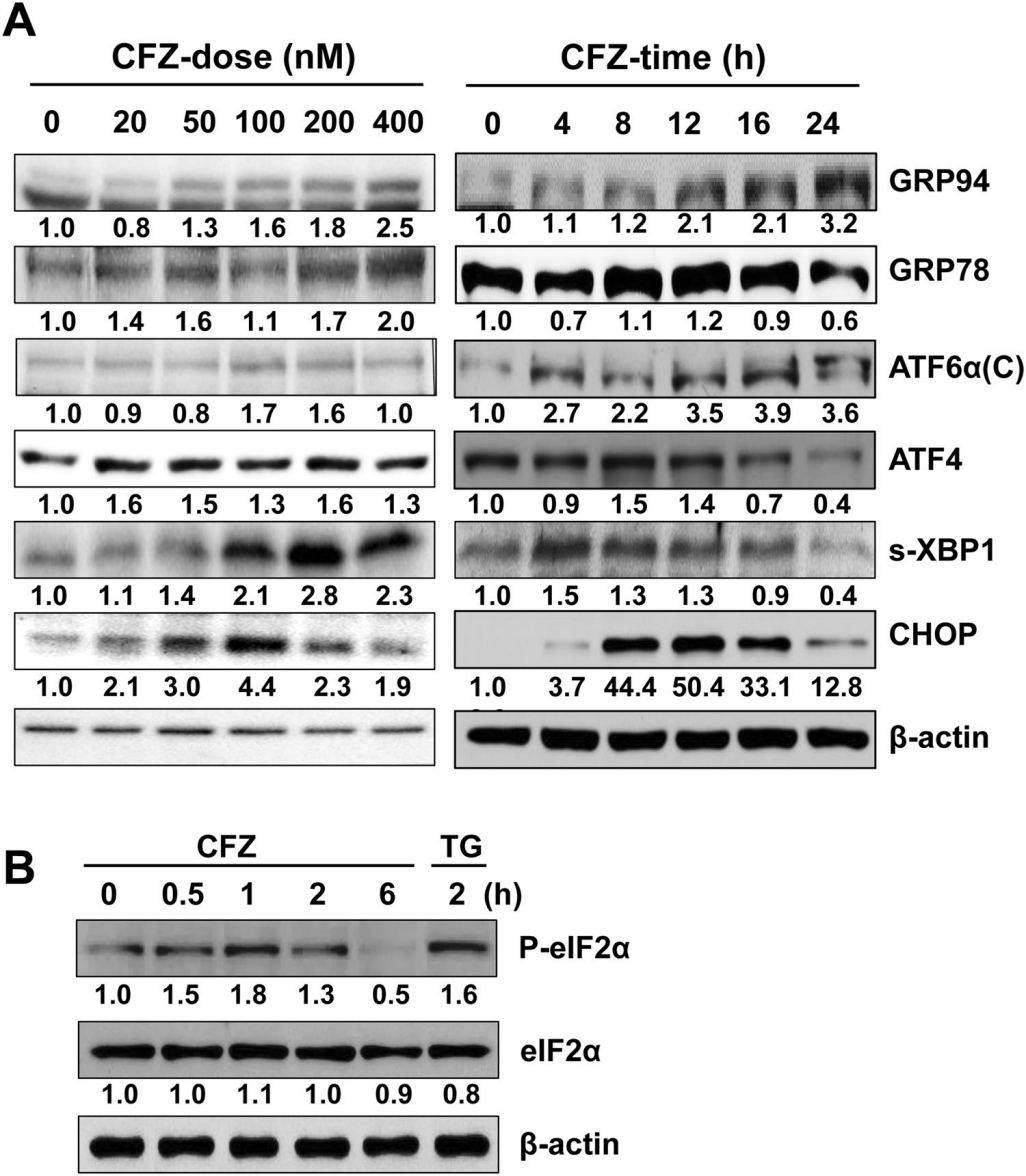
Effect of CFZ on the expression and activation of ER stress-associated proteins in SK-N-BE(2)-M17 cells. (**A**) Cells were treated with various concentrations (20–400 nM) of CFZ for 24 h (CFZ-dose) or treated with 200 nM CFZ for the indicated durations (CFZ-time). (**B**) Cells were treated with 200 nM CFZ for 0.5–2 h. Cell lysates from (**A**,**B**) were prepared and resolved by SDS-PAGE. Changes in the levels of ER stress-associated proteins were examined by western blot analysis with antibodies against GRP94, GRP78, cleaved ATF6α, ATF4, XBP1, CHOP, P-eIF2α, eIF2α, and β-actin. Results shown are representative of those obtained in more than three independent experiments.

To confirm our hypothesis that CFZ induces apoptosis via ER stress, the levels of ER stress-associated proteins, such as GRP94, GRP78, ATF6α, ATF4, s-XBP1, CHOP, eIF2α, and phosphorylated eIF2α (P-eIF2α), were examined by western blot analysis. Various concentrations of CFZ at different time points were used. As shown in Fig. 3A, GRP94, GRP78, ATF6α, and s-XBP1 levels were especially increased by CFZ treatment at higher concentrations (200–400 nM), whereas ATF4 and CHOP were expressed when cells were treated with low concentrations (20–100 nM) of CFZ. Interestingly, CHOP and s-XBP1 levels increased gradually and reached maximum at 100 and 200 nM CFZ, respectively. However, high concentrations of CFZ did not increase the expression of these proteins. The time-dependent expression patterns also varied with proteins. The levels of GRP94 and ATF6α increased gradually until the late time point (24 h), whereas those of GRP78, ATF4, s-XBP1 and CHOP were transient, with maximum levels at 8–12 h after treatment with 200 nM CFZ (Fig. 3A). Furthermore, P-eIF2α level increased at early time points (0.5–2 h) with maximum at 1 h when cells were treated with 200 nM CFZ (Fig. 3B). The level of P-eIF2α induced by CFZ was comparable to that induced by thapsigargin (TG), a representative ER stress inducer. Although the dose- and time-dependent expression patterns were slightly different, most ER stress-associated protein levels increased in CFZ-treated cells. These results suggest that CFZ can induce ER stress, which might play a role in CFZ-induced apoptosis in SK-N-BE(2)-M17 cells.

### CFZ increases mitochondrial damage, ROS production, and autophagosome formation in SK-N-BE(2)-M17 cells

**Figure 4:**
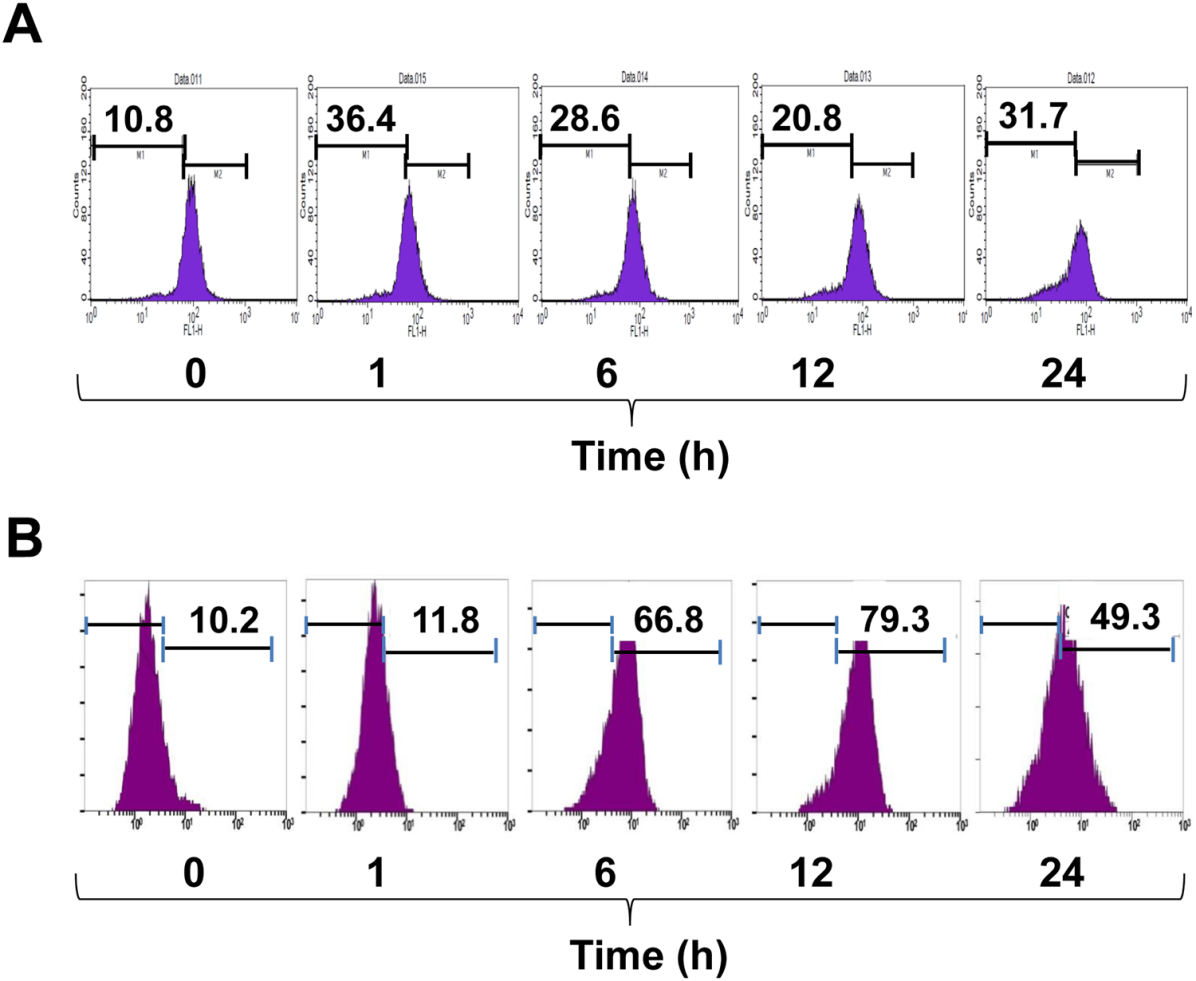
Effects of CFZ on MMP loss and ROS production in SK-N-BE(2)-M17 cells. SK-N-BE(2)-M17 cells were treated with vehicle or 200 nM CFZ for 1, 6, 12 and 24 h. The cells were then stained with either 10 μM DiOC_6_ for 15 min (**A**) or 25 μM DCFH-DA for 30 min (**B**) and the fluorescence intensity was measured by flow cytometry. Data on the marked region indicate the percentage of cells with MMP loss (**A**) and ROS production (**B**).

Mitochondrial damage and dysfunction are closely related to ER stress and apoptotic cell death. Mitochondrial membrane potential (MMP) loss is usually an indicator of mitochondrial damage and dysfunction. To measure the effect of CFZ on MMP loss, cells were treated with 200 nM CFZ for 1, 6, 12, and 24 h. Cells were then labeled with DiOC6 for 15 min to detect MMP using flow cytometry. As shown in Fig. 4A, CFZ increased MMP loss during the experimental period with biphasic peaks at an early time point (1 h) as well as at a later time point (24 h). CFZ treatment increased the ratio of cells with low fluorescence (in the left region) from 10.8% to 36.4% at 1 h, 28.6% at 6 h, 20.8% at 12 h, and 31.7% at 24 h (Fig. 4A). Thus, CFZ induced mitochondrial damage and MMP loss during the experimental periods (1–24 h) in SK-N-BE(2)-M17 cells.

Previously, it has been suggested that ROS-induced oxidative stress cross-talks with ER stress and mitochondrial damage, resulting in apoptotic cell death^23^. Since CFZ induced activation of caspase-4 and 9, we postulated that ROS might be involved in CFZ-induced mitochondrial damage as well as ER stress. To investigate whether CFZ can induce ROS production, cells were treated with 200 nM CFZ for 1, 6, 12, and 24 h, followed by treatment with DCFH-DA for 30 min and measurement of ROS-induced fluorescence by flow cytometry. Data showed that CFZ increased ROS production, as evaluated by the higher numbers of fluorescent cells (in the right regions): from 10.2% to 11.8% at 1 h, 66.8% at 6 h, 79.3% at 12 h, and 49.3% at 24 h in CFZ-treated cells (Fig. 4B). The ROS accumulation might play a role in caspase activation and apoptotic cell death.

**Figure 5:**
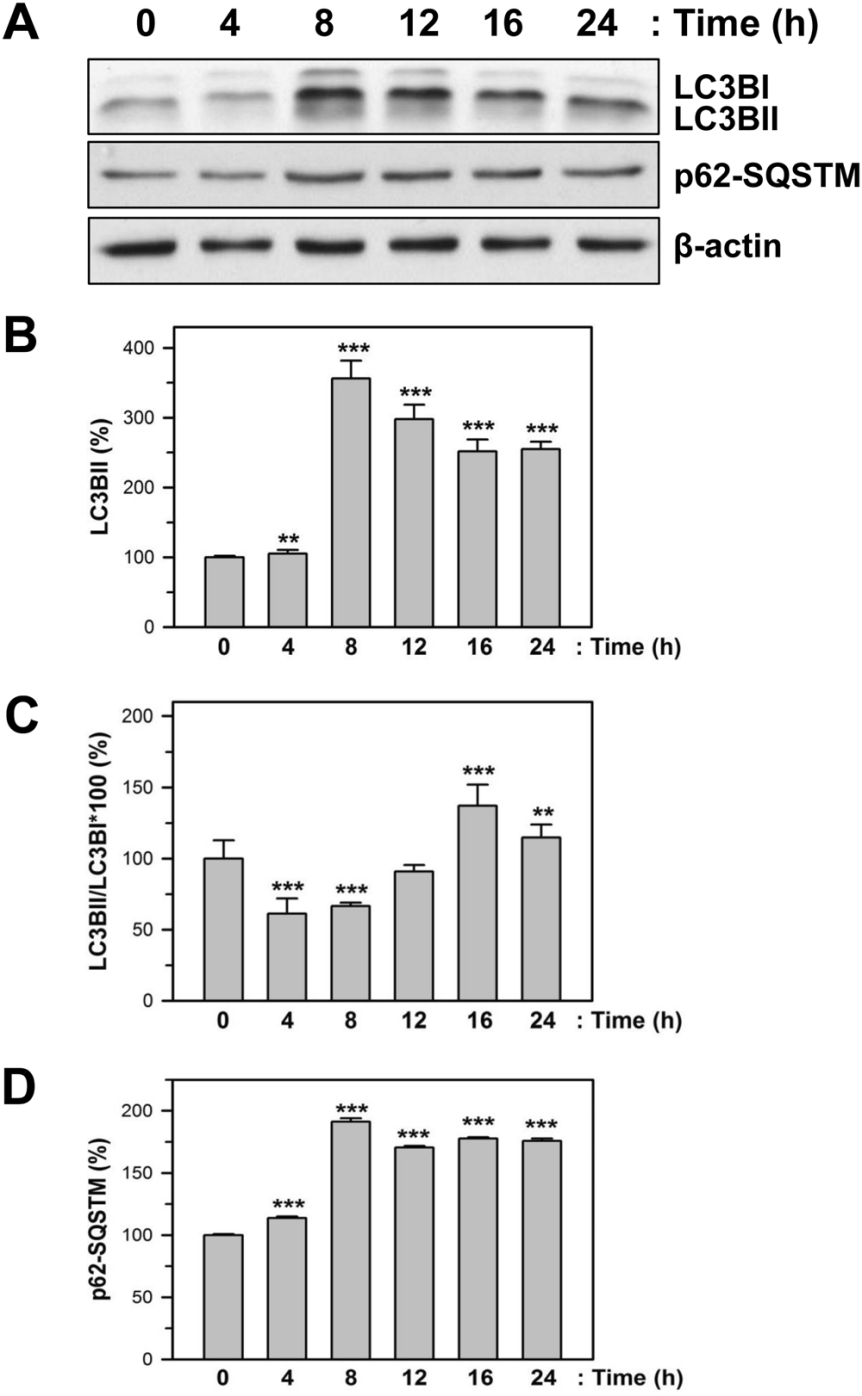
Effect of CFZ on autophagy markers in SK-N-BE(2)-M17 cells. (**A**) SK-N-BE(2)-M17 cells were treated with 200 nM CFZ for the indicated times. Total cell extracts were analyzed by SDS-PAGE and western blot with antibodies against autophagy marker proteins, such as LC3BI/II, p62-SQSTM, and β-actin. (**B–D**) The band density was analyzed by densitometry of each band and plotted as the relative level (%) of LC3BII (B) and p62-SQSTM (D), the ratio of LC3BII/LC3BI (C). **P < 0.01 and ***P < 0.001, compared to the vehicle-treated control.

The negative role of autophagy in apoptosis has been suggested in various apoptotic conditions. The expression and cleavage of LC3B increased with the progression of autophagy. To determine the effect of CFZ on autophagosome formation and subsequent autophagy progression, SK-N-BE(2)-M17 cells were treated with 200 nM CFZ for the indicated durations and the expression of autophagy markers, LC3B and p62/SQSTM, was examined by western blot analysis. Results showed that the levels of both LC3BI (intact form) and LC3BII (cleaved form), and p62/SQSTM were increased by CFZ treatment at 8–24 h in SK-N-BE(2)-M17 cells (Fig. 5A). Since the subsequent degradation of LC3BI and II was observed during the late time points (16–24 h) (Fig. 5A,B), CFZ probably induce subsequent autophagy progression. However, the ratio of LC3BII/LC3BI was high at 16–24 h (Fig. 5C) and the degradation of p62/SQSTM did not occur at the late time points (Fig. 5D), indicating an inefficient autolysosomal degradation. Our results suggest that CFZ can induce autophagosome formation and inefficient autolysosomal degradation.

### An antioxidant N-acetylcysteine reduces CFZ-induced cytotoxicity in SK-N-BE(2)-M17 cells

**Figure 6:**
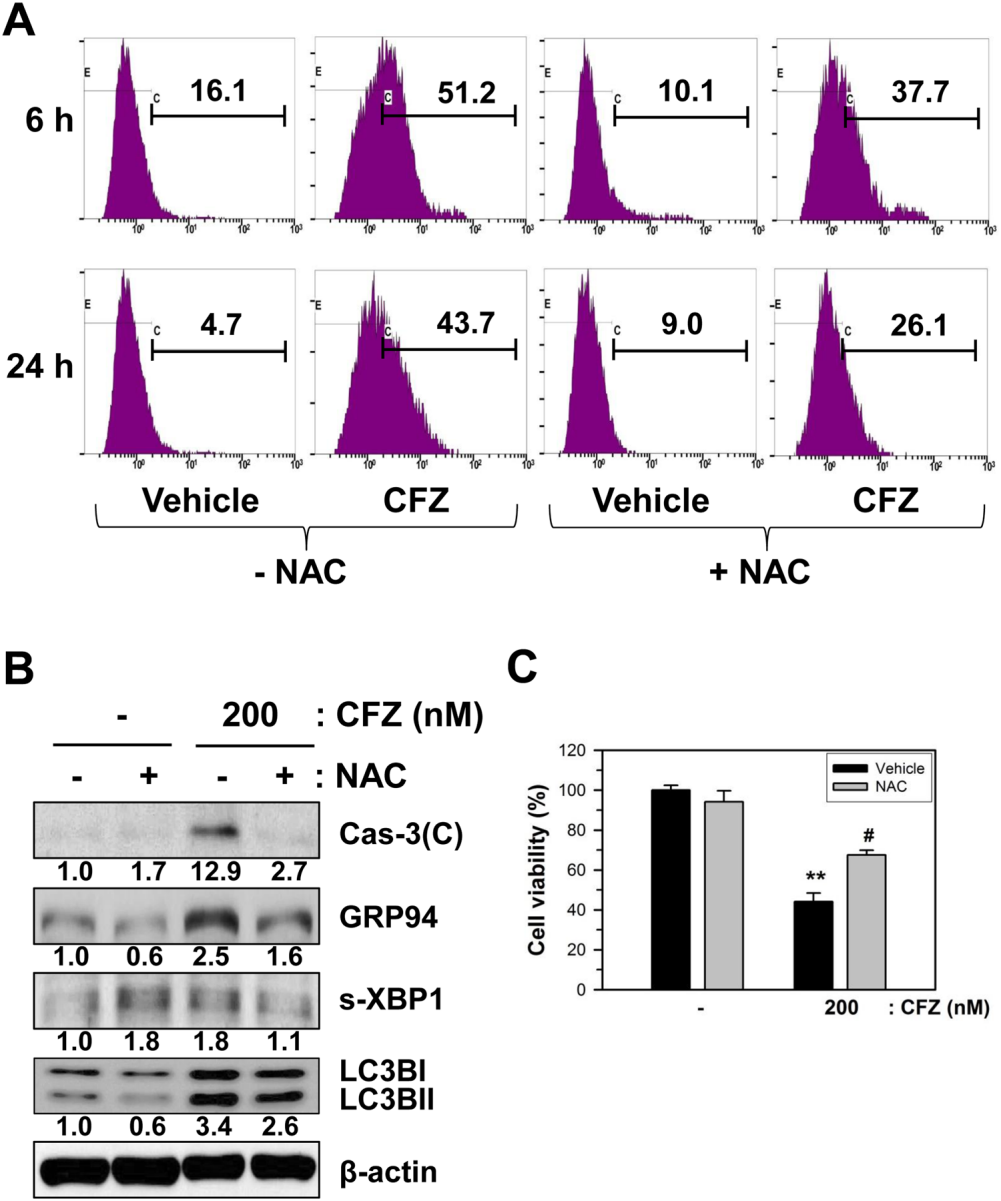
Effects of NAC on CFZ-induced ROS production, cell viability, and death-associated proteins in SK-N-BE(2)-M17 cells. (**A**) To measure ROS production, cells were pretreated with vehicle or 5 mM NAC for 1 h before treatment with 200 nM CFZ in SK-N-BE(2)-M17 cells. After 6 and 24 h treatment with vehicle or CFZ in the presence of NAC, cells were stained with 25 μM DCFH-DA for 30 min. The stained cells were analyzed by flow cytometry. (**B**) The NAC-pretreated cells were treated with 200 nM CFZ and the cell extracts were analyzed by western blotting with antibodies against cleaved caspase-3, GRP94, XBP1, LC3BI/II, and β-actin. (**C**) Cells were pretreated with vehicle (black bar) or 5 mM NAC (gray bar) for 1 h before treatment with CFZ (200 nM) in SK-N-BE(2)-M17 cells. Cell viability was measured by the MTT assay. The percentage cell viabilities were plotted.

Since CFZ increased ROS production, we examined whether ROS production plays an important role in CFZ-induced apoptosis using an antioxidant N-acetylcysteine (NAC). SK-N-BE(2)-M17 cells were preincubated with 5 mM NAC for 1 h and then ROS production was measured as described in Materials and Methods. NAC by itself slightly altered ROS generation from 16.1% to 10.1% at 6 h and from 4.7% to 9.0% at 24 h. However, NAC reduced CFZ-induced ROS generation from 51.2% to 37.7% at 6 h and from 43.7% to 26.1% at 24 h (Fig. 6A).

NAC treatment reduced CFZ-induced increase in death-associated proteins, including caspase-3, GRP94, s-XBP1, and LC3BI/II (Fig. 6B). Evaluation of cell viability by MTT assay showed that NAC was able to significantly (P < 0.05) enhance the viability of CFZ-treated cells at a concentration of 200 nM (Fig. 6C). The results indicate that ROS might play a role in CFZ-induced cell death.

### CFZ and Cis additively induce cell cycle arrest and apoptotic cell death

**Figure 7:**
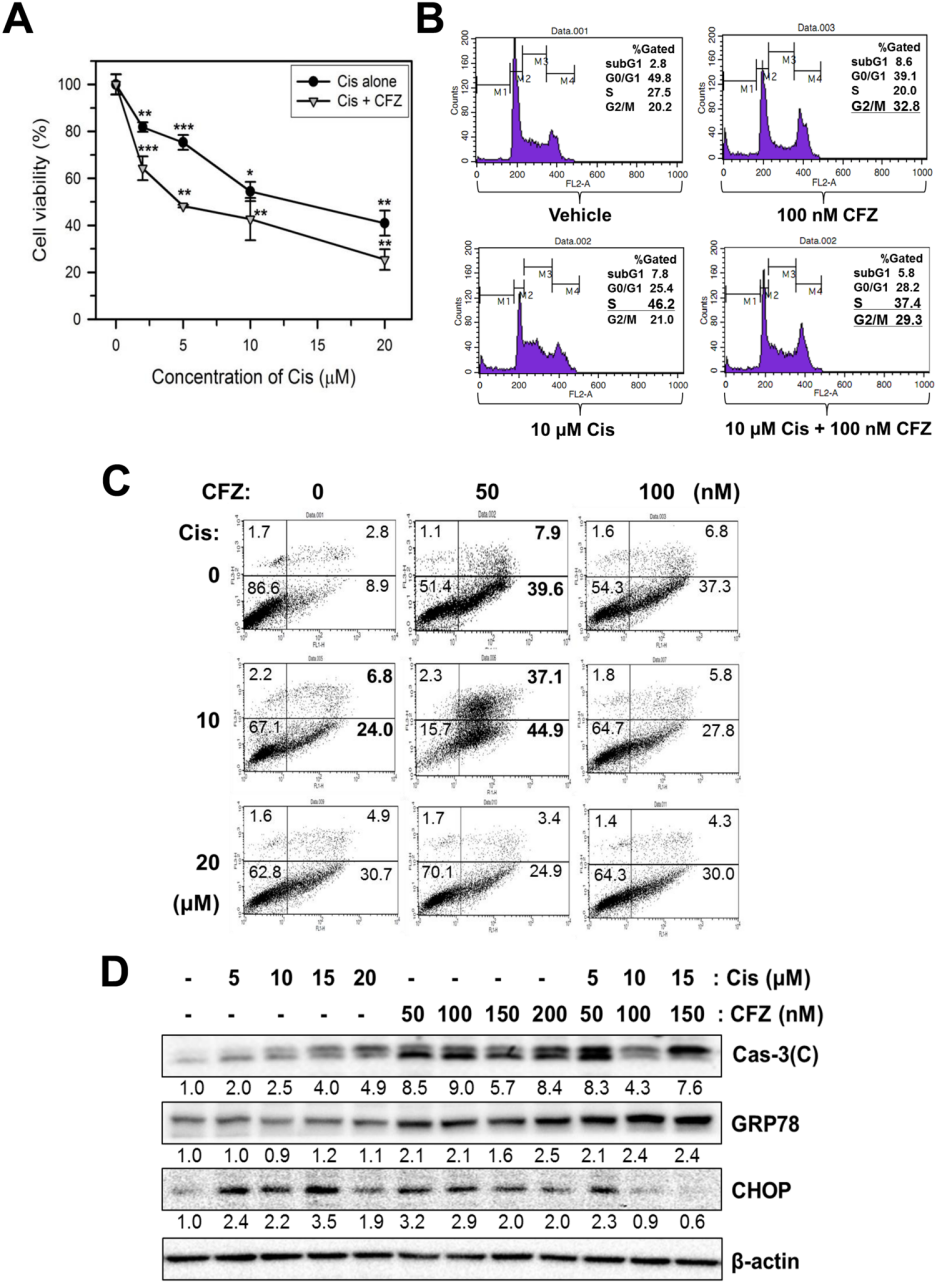
Additive effects of Cis and CFZ on cell viability, cell cycle arrest, and apoptosis in SK-N-BE(2)-M17 cells. (**A**) SK-N-BE(2)-M17 cells were treated with various concentrations (2–20 μM) of Cis in the absence (vehicle) or presence of 50 nM CFZ for 24 h. Cell viability was measured by the MTT assay. The percent cell viabilities are plotted. *P < 0.05, **P < 0.01, and ***P < 0.001, compared to the vehicle-treated control. (**B**) To examine cell cycle arrest, cells were treated with vehicle, 100 nM CFZ, 10 μM Cis, or 100 nM CFZ +10 μM Cis for 24 h. (**C**) Cells were treated with 50–100 nM CFZ, 10–20 μM Cis, or various combinations of CFZ + Cis. The cells were then stained with PI and annexin V-FITC, and apoptotic cells were evaluated by flow cytometry. (**D**) CFZ alone- or Cis alone-treated or co-treated cells were lysed and the cell extracts were analyzed by western blotting with antibodies against cleaved caspase-3, GRP78, CHOP, and β-actin.

Although Cis is used as a chemotherapeutic drug for treating various cancers, it also induces severe side effects, such as neurotoxicity, renal toxicity, and bone marrow suppression^33^. Therefore, reduction of its dosage might be necessary to reduce the side effects. To test whether CFZ can be used in a combination therapy with Cis, SK-N-BE(2)-M17 cells were co-treated with Cis and CFZ, and cell viability was examined using the MTT assay. The Cis treatment-induced dose-dependent reduction in cell viability was compared with that induced by co-treatment with 50 nM CFZ for 24 h. Co-treatment with CFZ and Cis additively reduced cell viability (Fig. 7A). The IC_50_ value of Cis was 13.6 μM in the absence of CFZ but 4.6 μM in the presence of CFZ. The additive effect of CFZ was significant at lower concentrations of Cis (P < 0.05 at 2 μM and P < 0.01 at 5 μM), whereas it was reduced at higher concentrations (10–20 μM) of Cis.

Previous studies revealed that CFZ caused cell cycle arrest at the G2/M phase (Fig. 2A); however, Cis caused cell cycle arrest in the S phase as previously reported^37^. When cell cycle arrest was examined after co-treatment of cells with 10 μM Cis and 100 nM CFZ, cell cycle arrest at both S and G2/M fraction was observed (Fig. 7B). In addition, changes in the ratio of apoptotic cells were also observed using PI/annexin V-FITC double staining. The percentage of early apoptotic cells increased from 8.9% to 44.9% in co-treated cells, compared to 39.6% in cells treated with 50 nM CFZ alone and 24.0% in cells treated with 10 μM Cis alone (Fig. 7C). The proportion of late apoptotic cells increased from 2.8% to 37.1% in the co-treated cells, compared to 7.9% in cells treated with 50 nM CFZ alone and 6.8% in cells treated with 10 μM Cis alone (Fig. 7C). The additive effect was absent in cells treated with higher concentrations of either CFZ (100 nM) or Cis (20 μM).

The effect of CFZ and Cis co-treatment on the levels of certain death-associated proteins was investigated by western blot analysis. Co-treatment with CFZ and Cis resulted in additive changes in the levels of cleaved caspase-3 and GRP78 (Fig. 7D). In contrast, the level of CHOP was reduced by the co-treatment. Previously, the dose-dependent alterations of CHOP had indicated that higher doses of CFZ (Fig. 3) reduced CHOP expression. Therefore, reduction of CHOP by the co-treatment might be because of higher combined dose of the two drugs.

### CFZ alone or in combination with Cis reduces cell viability in Cis-resistant SK-N-BE(2)-M17 cells

**Figure 8:**
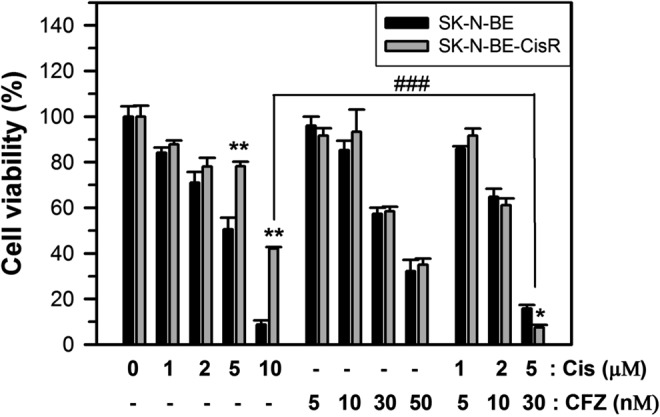
Changes in cell viability by CFZ treatment in Cis-resistant SK-N-BE(2)-M17 cells. Cis-resistant SK-N-BE(2)-M17 cells were prepared by treatment with 2 μM Cis for 20 days or longer in the 10% FBS culture media. The culture medium was refreshed after every 2 days. Control non-resistant cells and Cis-resistant cells were treated with various concentrations of Cis alone (1–10 μM), CFZ alone (5–50 nM), or various combinations of CFZ + Cis for 48 h. Cell viability was analyzed by the MTT assay and the percentage cell viabilities were plotted. **P < 0.01, compared to the vehicle-treated control.

Cis therapy is well-known to cause resistance, which results in treatment failure^34^. Therefore, we determined whether CFZ can be used in Cis-resistant cells. Cis-resistant cells were prepared from the surviving cell fractions after long-term treatment with 2 μM Cis (for 20 days or longer). MTT assay was performed to determine cell viability in CFZ alone- or Cis-alone treated or co-treated control cells and Cis-resistant cells. As expected, the effect of Cis treatment alone on cell viability was reduced in Cis-resistant cells, compared to that in control cells (Fig. 8), and high dose (10 μM) of Cis overcame this resistance. However, CFZ treatment reduced cell viability to similar extents in normal and Cis-resistant cells (Fig. 8). In addition, co-treatment with CFZ and Cis showed an additive effect. These results suggest that CFZ can induce cell death in Cis-resistant neuroblastoma cells.

## Discussion

Neuroblastoma is the most common childhood extracranial neoplasm, which accounts for approximately 15% of cancer-related deaths in children^38–40^. Although patients with low- and intermediate-risk neuroblastoma have demonstrated good treatment outcomes, the survivability for patients with high-risk neuroblastoma remains poor. Since chemotherapy before surgery improves the survivability of patients with neuroblastoma^41^, the development of better and specific chemotherapy for the treatment of high-risk neuroblastoma is required. Therefore, the present study investigated the possibility that proteasome inhibition by CFZ, a second-generation proteasome inhibitor without side effects, is a potential therapeutic strategy for the treatment of patients with neuroblastoma.

Since CFZ has been approved by the FDA for the treatment of multiple myeloma^11^, several laboratories have evaluated whether CFZ could be used for the treatment of other solid cancers, such as lung, colon, pancreas, breast, and head and neck cancer. Indeed, CFZ treatment significantly increased the overall survival of mice with metastatic cancer, without significant adverse drug reactions^14,42^. In the present study, morphological changes, detachment, and cell viability reduction were observed after treatment of SK-N-BE(2)-M17 neuroblastoma cells with CFZ (Fig. 1). Investigation of PI-stained and PI/annexin V-FITC double-stained cells using flow cytometry also revealed that CFZ reduces cancer cell viability by inducing cell cycle arrest at the G2/M phase (Fig. 2A) and apoptotic cell death (Fig. 2B). This observation of cell cycle arrest was in agreement with those of previous reports with other cell types. CFZ was reported to arrest cells at the G2/M phase in T-leukemia^43^, endometrial cancer^44^, and thyroid cancer cells^42^. These observations support our hypothesis that CFZ can be used as an anticancer drug for treating neuroblastoma.

Although several mechanisms of CFZ action have been suggested, they depended on the cell type being studied^14^. Apoptosis can be induced by the cleaved and activated caspases. In this study, we confirmed the activation of caspase-8, 9, 4, and 3 by CFZ treatments in SK-N-BE(2)-M17 neuroblastoma cells (Fig. 2C). The effect of CFZ on caspases in neuroblastoma cells was slightly different from that in chronic lymphocytic leukemia^45^ and mantle cell lymphoma^13^. In our experiment, caspase-8, 9, and 4 were activated earlier than caspase-3 after CFZ treatment. Activations of caspase-8 and 9 indicated implications of both extrinsic and intrinsic apoptosis pathways. In addition, caspase-4 activation confirmed that CFZ can induce ER stress, which may play a role in apoptosis induction by this drug in SK-N-BE(2)-M17 cells.

ER stress due to accumulation and aggregation of ubiquitinated/unfolded proteins has been suggested as one of the main mechanisms of apoptosis induction by proteasome inhibitors^14^. The dose- and time-dependent expressions of some ER stress-associated proteins were observed in CFZ-treated cells (Fig. 3). The time-dependent cleavage of ATF6α was biphasic with first induction at the early time points (4 h) and second induction at the late time points (12–24 h) after treatment with 200 nM CFZ. The expression of GRP94 showed a gradual increase until 24 h. The levels of ATF4, s-XBP1, and CHOP increased until a certain time point or concentration and then reduced until 24 h. Although the dose- and time-dependent effects of CFZ on ER stress-associated proteins are slightly different, depending on the cell types, ER stress seems to be associated with CFZ-induced apoptotic cell death in SK-N-BE(2)-M17 cells.

In cancer cells, autophagy is known to be important for cell viability and apoptosis. For example, CFZ and ONX 0912 co-treatment induced apoptosis as well as autophagy in HNSCC cells^46^. Autophagy induction might be caused by UPR and subsequent ATF4 upregulation, which might play a pro-survival role. Changes in the expression of autophagy markers indicated that the levels of both LC3BI and II increased at 8–12 h in CFZ-treated cells (Fig. 5). The p62/SQSTM levels were increased similarly by CFZ. The expression pattern of these autophagy markers in CFZ-treated cells suggests that CFZ can increase autophagosome formation, but it may not efficiently induce subsequent autophagy progression via lysosomal degradation. Autophagosome formation and autophagy induction by CFZ have been also reported by other researchers in different tumor cells^47,48^. The role of autophagosome formation and final lysosomal degradation in apoptosis has not yet been clarified. Further studies with different types of inhibitors and molecular modulations are required.

ROS have been shown to affect ER stress and mitochondrial dysfunction, and DNA damage, leading to apoptotic cell death. ROS production by CFZ has already been demonstrated in multiple myeloma cells^49^. Since caspase-9 was activated by CFZ (Fig. 2C), we postulated that ROS production and mitochondrial damage may be implicated in CFZ-induced apoptotic cell death in SK-N-BE(2)-M17 neuroblastoma cells. As expected, CFZ produced ROS and caused mitochondrial damage (Fig. 4). By treating cells with antioxidant NAC to remove ROS, we confirmed that ROS production was involved in cell viability reduction, ER stress, autophagosome formation, and caspase-3 activation (Fig. 6). CFZ-induced oxidative stress might cause the mitochondrial damage and MMP loss, which play a partial role in apoptosis.

Cis has been used for treating various cancers as it can damage cancer cells via apoptosis^33^. However, Cis also damages normal cells, which leads to toxic side effects^33^. In addition, long-term treatment of cancer cells with Cis results in Cis resistance^34^. Therefore, strategies should be developed to reduce the side effects and to induce chemosensitization in Cis-resistant cells. One approach involved combining Cis with other chemotherapeutic drugs, which has been widely used in treatment of cancer^30,33^. The present study showed that the effects of CFZ on cell cycle arrest and apoptosis were additive to those of Cis in SK-N-BE(2)-M17 human neuroblastoma cells (Fig. 7). Since CFZ induced cell cycle arrest at the G2/M phase but Cis induced it at the S phase (Figs 2 and 7), co-treatment of CFZ with Cis resulted in both G2/M and S phase arrest, and enhanced caspase-3 activation and ER stress responses (Fig. 7). In addition, similar additive effects of CFZ were observed in Cis alone-treated control and Cis-resistant neuroblastoma cells (Fig. 8). Interestingly, when SHP77 small cell lung cancer (SCLC) cells were treated with CFZ + Cis, additive or synergistic antitumor effect was not observed^11,14^. However, the present data strongly suggest that a combination of low doses of Cis and CFZ produced beneficial effects in neuroblastoma cells (Fig. 7), suggesting that co-treatment with Cis and CFZ at low doses might be a good treatment strategy for patients with neuroblastoma.

In conclusion, CFZ can induce cell cycle arrest and apoptosis through ER stress, ROS production, and mitochondrial dysfunction in SK-N-BE(2)-M17 neuroblastoma cells. As the effects of CFZ and Cis are additive in terms of cell cycle arrest, apoptotic cell death, and ER stress, co-treatment of CFZ with Cis might reduce the individual dosages of CFZ and Cis, resulting in lower side effects and overcoming of drug resistance. Therefore, a combination therapy with CFZ and Cis might be beneficial for the treatment of neuroblastoma, a cancerous tumor that begins in the nerve tissue of infants and young children.

## Materials and Methods

### Materials

Dulbecco’s modified Eagle’s medium (DMEM) and fetal bovine serum (FBS) were purchased from Corning (Corning, NY, USA). Trypsin-EDTA, and antibiotic-antimycotic agents were purchased from Gibco (Grand lsland, NY, USA). Antibodies specific for caspase-3, 4, 8, and 9, phospho-Ser^51^-elF2α, and LC3B were purchased from Cell Signaling (Beverly, MA, USA). Antibodies specific for CHOP/GADD153, GRP94, GRP78, ATF6α, elF2α, and XBP1 were purchased from Santa Cruz Biotechnology (Santa Cruz, CA, USA). Antibodies specific for ATF4 was obtained from Abcam (Cambrige, CA, USA) and antibodies specific for p62/SQSTM was from Proteintech (Chicago, IL, USA). Horseradish peroxidase (HRP)-conjugated anti-rabbit and anti-mouse secondary antibodies were purchased from Vector Laboratories (Burlingame, CA, USA). Dimethyl sulfoxide (DMSO), N-acetyl-l-cysteine (NAC), propidium iodide (PI), DNase free-RNase A, 2′,7′-dichlorodihydrofluorescein diacetate (DCFH-DA), 3,3′-dihexyloxacarbocyanine iodide (DiOC6), Cis, thapsigargin (TG), and antibodies specific for β-actin were purchased from Sigma-Aldrich (St. Louis, MO, USA). FITC/annexin V apoptosis detection kit I was purchased from BD Biosciences (San Diego, CA, USA). CFZ was purchased from Biovision (Mountain View, CA, USA); 3-(4–5-dimethylthiazol-2-yl)-2,5-diphenyltetrazoluim bromide (MTT) was from Gold Biotechnology (Olivette, MO, USA); enhanced chemiluminescence (ECL) detection kit, polyvinylidene fluoride (PVDF) membrane, and nitrocellulose (NC) membrane from GE Healthcare (Fairfield, CT, USA); Bradford protein assay dye reagent and 30% acrylamide/bis solution was from BioRad (Hercules, CA, USA).

### Cell culture

SK-N-BE(2)-M17 (male), SH-SY5Y (female), and Neuro-2A mouse neuroblastoma cells were purchased from American Type Culture Collection (Manassas, VA, USA), and IMR-32 (male), SK-N-SH (female) and SK-N-MC (female) human neuroblastoma cells were obtained from Korean Cell Line Bank (Seoul, Korea). All cells except IMR-32 were cultured in DMEM supplemented with L-glutamine (300 mg/ml), 25 mM HEPES, 25 mM NaHCO_3_, 10% heat-inactivated FBS and antibiotic-antimycotic agents at 37 °C in a 5% CO_2_ incubator. IMR-32 cells were grown in RPMI media supplemented with L-glutamine (300 mg/ml), 25 mM HEPES, 25 mM NaHCO_3_, 10% heat-inactivated FBS and antibiotic-antimycotic agents. Before treatment with CFZ, Cis, or other drugs, cells were refreshed with the culture media and stabilized for 3 h.

### Measurement of cell viability by MTT assay

Cell viability was determined using the MTT assay. For the MTT assay, cells were seeded in a 24-well plate at a density of 2 × 10^4^ cells/ml and incubated for 1 day. Cells were then treated with various concentrations of CFZ or Cis alone, or CFZ + Cis for 24 or 48 h. After incubation, fifty microliter of MTT stock solution (5 mg/ml) was added to each well. After incubation for 2 h, 200 μl DMSO was added to dissolve the formazan precipitates. The completely dissolved formazan was transferred to a 96-well plate. The amount of formazan salts was detected by measuring the optical density (OD) at 570 nm using an enzyme-linked immunosorbent assay (ELISA) plate reader (VICTOR^3^, PerkinElmer Life and Analytical Sciences, Turku, Finland). The numerical OD value was quantified as a percentage of the vehicle-treated control.

### Cell cycle analysis

Cells were seeded in a 6-well plate at a density of 2 × 10^5^ cells per well and incubated for 1 day. Cells were treated with CFZ or Cis alone, or CFZ + Cis for 24 h in 10% FBS-DMEM. After detached cells in the medium were transferred to a 15-ml ice-cold Falcon tube, remaining cells in the culture dish were harvested by trypsinization, washed with 1 × PBS, and combined with the detached cells in the 15-ml Falcon tube. Cells were collected by centrifugation and fixed in 70% ethanol overnight at 4 °C. Fixed cells were collected by centrifugation and washed with ice-cold 1 × PBS. Cells were then incubated with DNase-free RNase A and PI solution for 30 min at 37 °C. The stained cells were kept on ice in the dark and analyzed using FACSCalibur flow cytometer (San Diego, CA, USA) with FL2 channel. Ten thousand events were analyzed per sample.

### PI/Annexin V-FITC double staining

Apoptosis was determined by double-staining of intact cells with both PI and annexin V-FITC. For the double-staining experiment, cells were seeded in a 6-well plate at a density of 2 × 10^5^ cells per well and incubated for 1 day. Cells were then treated with various concentrations of CFZ or Cis alone, or CFZ + Cis in 10% FBS-containing media for 24 h. Media with detached cells were transferred to a 15 ml ice-cold Falcon tube and stored on ice. The culture dishes were washed with 1 × PBS and treated briefly with 1 × trypsin-EDTA to harvest cells. Cells harvested by centrifugation were suspended with 1 × binding buffer and incubated with PI and annexin V-FITC reagent at room temperature in the dark for 15 min. The stained cells were kept on ice in the dark and analyzed by flow cytometry using a FACSCalibur flow cytometer with FL1 and FL2 channels or FL1 and FL3 channels. Ten thousand events were analyzed per sample.

### Western blot analysis

Total cell extracts were prepared by treating cells with RIPA buffer (50 mM Tris base, 150 mM NaCl, 2 mM EDTA, 1 mM EGTA, 1 mM Na_3_VO_4_, 10 mM NaF, 1% IGEPAL, 0.1% sodium sodecyl sulfate (SDS), 0.5% sodium deoxycholate) containing 0.5% protease and phosphatase inhibitor cocktail. Lysates were clarified by centrifugation and the protein concentration of the supernatant was determined using the Bradford protein assay reagent. Twenty micrograms of total cell lysates were denatured in 1 × SDS loading dye containing dithiothreitol. For western blot analysis, equal amounts of proteins were resolved by 10% SDS-polyacrylamide gel electrophoresis and analyzed. Proteins on the gel were transferred onto a PVDF membrane. Each membrane was blocked for 1 h with 5% skim milk in Tris-buffered saline with 0.05% Tween 20 (TBST). The membranes were then incubated with 1:500–1,000 dilutions of primary antibodies in the blocking buffer, followed by incubation with 1:1,000 dilution of HRP-conjugated secondary antibodies with activity in TBST for 1 h at room temperature. The immune complexes with specific proteins were visualized using an ECL detection kit according to the manufacturer’s protocol. The protein bands were analyzed by densitometry using a Bio-Rad image analyzer (ChemiDoc MP, Hercules, CA, USA). Equal protein loading was assessed by normalization against the density of the β-actin band in the corresponding lane.

### Measurement of ROS production

To measure the level of ROS, cells were seeded in a 6-well plate at a density of 2 × 10^5^ cells per well and incubated for 1 day. Cells were then treated with CFZ for the indicated times. After incubation, the cells were washed with 1 × PBS and culture media containing 25 μM DCFH-DA was added to each well. After incubation for 30 min at 37 °C, the cells were washed with 1 × PBS and treated with 1 × trypsin-EDTA for 1–2 min. Cells were harvested by centrifugation. The collected cells were washed twice with ice-cold 1 × PBS and the fluorescence intensity was measured using a FACSCalibur flow cytometer with excitation and emission wavelengths of 488 and 525 nm, respectively. Ten thousand events were analyzed per sample.

### Measurement of mitochondrial membrane potential

To measure MMP, cells were seeded and incubated for 1 day, followed by treatment with CFZ for the indicated times. After incubation, cells were washed with 1 × PBS and culture medium containing 10 μM DiOC_6_ was added to each well. After incubation for 15 min at 37 °C, the cells were washed with 1 × PBS and treated with 1 × trypsin-EDTA for 1–2 min. Cells were harvested by centrifugation. The collected cells were washed twice with ice-cold 1 × PBS and the fluorescence intensity was measured using a FACSCalibur flow cytometer with excitation and emission wavelengths of 482 and 504 nm, respectively. Ten thousand events were analyzed per sample.

### Statistical analysis

All data are presented as the mean ± standard deviation of at least three independent experiments. Statistical comparisons were performed using one-way analysis of variance (ANOVA), followed by Student’s t-test using Microsoft Excel program (Microsoft Corp., San Diego, CA, USA). P values of < 0.05 were considered statistically significant.
